# Identification of *Dermacentor* *reticulatus* Ticks Carrying *Rickettsia* *raoultii* on Migrating Jackal, Denmark

**DOI:** 10.3201/eid2312.170919

**Published:** 2017-12

**Authors:** Kirstine Klitgaard, Mariann Chriél, Anastasia Isbrand, Tim K. Jensen, René Bødker

**Affiliations:** National Veterinary Institute, Lyngby, Denmark

**Keywords:** Long-distance migration, carnivores, ticks, bacteria, zoonoses, vector-borne infections, *Dermacentor reticulatus*, *Rickettsia raoultii*

## Abstract

From a migrating golden jackal (*Canis aureus*), we retrieved 21 live male *Dermacentor reticulatus* ticks, a species not previously reported from wildlife in Denmark. We identified *Rickettsia raoultii* from 18 (86%) of the ticks. This bacterium is associated with scalp eschar and neck lymphadenopathy after tick bite syndrome among humans.

Since 2012, Denmark has received a sudden and poorly understood wave of gray wolves (*Canis lupus*) and golden jackals (*C. aureus*) migrating long distances from their birthplaces in eastern and central Europe (*1*). These long-distance dispersals create a risk for introducing tick vectors and pathogens to new geographic areas. We report discovery of *Dermacentor reticulatus* ticks infected with *Rickettsia raoultii* from a wild golden jackal in Thorsminde, in the western region of Denmark.

## The Study

In February 2017, the wildlife service delivered the body of a golden jackal from Western Jutland, >200 km north of the Denmark–Germany border, to the National Veterinary Institute (Lyngby, Denmark). During necropsy, we collected 21 male *Dermacentor reticulatus* ticks, a species that had not previously been reported among wildlife in Denmark. We screened the ticks and a blood sample from the jackal for tickborne pathogens by using a high-throughput real-time PCR (*2*). This assay enables simultaneous detection of 37 European tickborne pathogens, including the spotted fever group (SFG) *Rickettsia*, and specifically the species *R. conorii, R. slovaca, R. massiliae*, and *R. helvetica*, as well as confirmation of 4 tick species, including *D. reticulatus* (*2*). Tick DNA was extracted as previously described (*3*).

PCR confirmed the morphologic characterization: all the ticks collected from the jackal were *D. reticulatus*. One tick contained *Anaplasma phagocytophilum*, and 18 ticks contained SFG *Rickettsia* spp.; however, the specific *Rickettsia* species was not among the 4 species included in the PCR. To identify the species, we PCR amplified the *ompA, ompB*, and *gltA* genes for a subset of 4 samples and sequenced them as previously described (*4*,*5*). For the remaining 14 samples, we sequenced the *ompA* gene. A BLAST search (http://www.ncbi.nlm.nih.gov/BLAST/) identified the *ompB* gene as 100% and the *ompA* and *gltA* genes as 99% identical to the genome sequence of the type strain of *Rickettsia raoultii* sp. nov. strain Khabarovsk^T^ (CSUR R3^T^, ATCC VR-1596^T^) (*6*). The specific primers used for gene amplification and sequencing of bacteria identified from *D. reticulatus* ticks by real-time PCR are provided in the Table. We deposited the sequences we obtained into GenBank (accession nos. MF166729–36 and MF166741–44).

**Table:** Primers used for gene amplification and sequencing of *Rickettsia* spp. obtained from *Dermacentor* *reticulatus* ticks from a migrating jackal, Denmark

Primer name	Primer sequence, 5′ → 3′	Target gene	Length, bp	Reference
120–2,788	AAACAATAATCAAGGTACTGT	*ompB*	765	(*5*)
120–3,599	TACTTCCGGTTACAGCAAAGT			
Rr 190.70p	ATGGCGAATATTTCTCCAAAA	*ompA*	631	(*4*)
Rr190–701n	GTTCCGTTAATGGCAGCATCT			
RpCS877p	GGGGACCTGCTCACGGCGG	*gltA*	380	(*4*)
RpCS1258n	ATTGCAAAAAGTACAGTGAACA			

The surge of large carnivores, reaching countries in northern Europe from breeding areas in central Europe, is a recent development that appears to be caused by reduced hunting resulting from effective wildlife protection (*1*,*7*). This migration may facilitate the spread of vectors and zoonotic pathogens into new regions. Even if these migrations do not result in the establishment of a new carnivore population, Denmark has high fox and deer densities in the forests, as well as farm animals grazing on pastures, that can support tick species such as *D. reticulatus* or *D. marginatus*.

*D. reticulatus* ticks are spreading rapidly through Europe, and changes in the environment, climate, or both seem to be favorable to the establishment of this tick in areas not previously supporting populations of the species (*8*). *D. reticulatus* ticks are established in the southern United Kingdom, and recently, also in the Netherlands. Both countries lack wolf and jackal populations (*9*). Therefore, it is the actual process of migration and not the establishment of the carnivores in Denmark that constitutes a risk.

However, for these migrations to result in the introduction of ticks, the migrations must first originate in *Dermacentor* tick–endemic areas, which are located several hundred kilometers south of the Danish border. Second, the migrations must be rapid enough for the ticks to remain attached to the migrating carnivores during the entire migration. Individual gray wolves are indeed capable of migrating distances of 800–1,200 km within a short period; the first wolf known to arrive in northern Jutland has been genetically traced to the border area between Germany, the Czech Republic, and Poland, >800 km away from Denmark (*1*).

The finding of 21 adult male *D. reticulatus* ticks on a jackal hunted in western Denmark strongly indicates that jackal migrations are also long enough to originate from *Dermacentor* tick–endemic areas and fast enough to allow the ticks to complete the migration to Denmark. The origin of the jackal received at the National Veterinary Institute is unknown, but the nearest known breeding populations of golden jackals are in Austria and Hungary (*7*). Female ticks were absent on the jackal because they drop off after feeding, but each female *D. reticulatus* tick can lay up to 7,200 eggs (*8*), which would have been deposited somewhere between the endemic tick region and Denmark. Some of those eggs may have landed in a favorable environment.

The SFG *Rickettsia R. slovaca* and *R. raoultii*, which are exotic to Denmark, are the most consequential zoonotic pathogens carried by *D. reticulatus* ticks. These bacteria are a public health concern because they cause scalp eschar and neck lymphadenopathy after tick bite (SENLAT) syndrome (*10*,*11*). SENLAT is an emerging tickborne infection and among the most common tickborne rickettsiosis in Europe (*8*). The disease is defined as the association of a tick bite, an inoculation eschar on the scalp, and cervical adenopathies (*10*,*11*). The main signs and symptoms are a crustaceous lesion (early) or eschar (late) at the site of the tick’s attachment and regional painful lymph nodes (*10*,*11*).

*R. raoultii* was only recently characterized and named from bacteria isolated from *Dermacentor* tick species collected in Russia and France (*6*). In 2016, *R. raoultii* was reported for the first time in Austria and the Czech Republic (*12*,*13*). A relatively high prevalence of *R. raoultii* has been reported in ticks from various regions of Europe, from 14.9% in Austria (*12*) to 58% in Hungary (*14*). In the case we report, the high prevalence (85.7%) of *R. raoultii*–infected ticks from the jackal may have been accentuated by co-feeding transmission between ticks on the same host (*8*).

We did not identify any rickettsiae from the blood of the jackal by PCR. However, because ticks are able to sustain rickettsial transmission cycles transovarially and transstadially, it is possible that there is, in fact, no host reservoir of *R. raoultii* (*8*). If the ticks serve as a reservoir of *R. raoultii*, the distribution of this bacterium will be identical to that of the tick’s area of dispersal (*15*). *D. reticulatus* males remain on the host for 2–3 months and have an intermittent feeding behavior (*8*). This trait makes them vectors of pathogenic agents, and recent studies have also shown that male *D. reticulatus* ticks play a strategic role in the transmission of *R. slovaca* and *R. raoultii* to humans (*8*).

Although SENLAT is a milder rickettsiosis, physicians should be aware of the potential diagnosis of this emerging tickborne disease. A reason for concern is that the intracellular *Rickettsia* infections require treatment with different antimicrobial drugs than, for example, *Borrelia* infections.

## Conclusions

The simultaneous finding of a new carnivore, a new tick vector, and a new zoonotic pathogen in Denmark demonstrates that the unexpected recent wave of large carnivores migrating over long distances into Denmark is more than a theoretical risk to human and animal health. *Ixodes ricinus* ticks are abundant in most forests in Denmark. However, forests cover only 14% of Denmark’s area, and the preference of *D. reticulatus* ticks for more open areas could dramatically increase the area of Denmark and northern Europe with a risk for tick bites and tickborne infections.

## References

[1] Andersen LW, Harms V, Caniglia R, Czarnomska SD, Fabbri E, Jędrzejewska B, et al. Long-distance dispersal of a wolf, *Canis lupus*, in northwestern Europe. Mammal Res. 2015;60:163–8. 10.1007/s13364-015-0220-6

[2] Michelet L, Delannoy S, Devillers E, Umhang G, Aspan A, Juremalm M, et al. High-throughput screening of tick-borne pathogens in Europe. Front Cell Infect Microbiol. 2014;4:103. 10.3389/fcimb.2014.00103 25120960 PMC4114295

[3] Jensen PM, Christoffersen CS, Moutailler S, Michelet L, Klitgaard K, Bødker R. Transmission differentials for multiple pathogens as inferred from their prevalence in larva, nymph and adult of *Ixodes ricinus* (Acari: *Ixodidae*). Exp Appl Acarol. 2017;71:171–82. 10.1007/s10493-017-0110-5 28255923

[4] Regnery RL, Spruill CL, Plikaytis BD. Genotypic identification of rickettsiae and estimation of intraspecies sequence divergence for portions of two rickettsial genes. J Bacteriol. 1991;173:1576–89. 10.1128/jb.173.5.1576-1589.1991 1671856 PMC207306

[5] Roux V, Fournier PE, Raoult D. Differentiation of spotted fever group rickettsiae by sequencing and analysis of restriction fragment length polymorphism of PCR-amplified DNA of the gene encoding the protein rOmpA. J Clin Microbiol. 1996;34:2058–65. 8862558 10.1128/jcm.34.9.2058-2065.1996 PMC229190

[6] Mediannikov O, Matsumoto K, Samoylenko I, Drancourt M, Roux V, Rydkina E, et al. *Rickettsia raoultii* sp. nov., a spotted fever group rickettsia associated with *Dermacentor* ticks in Europe and Russia. Int J Syst Evol Microbiol. 2008;58:1635–9. 10.1099/ijs.0.64952-0 18599708

[7] Trouwborst A, Krofel M, Linnell JDC. Legal implications of range expansions in a terrestrial carnivore: the case of the golden jackal (*Canis aureus*) in Europe. Biodivers Conserv. 2015;24:2593–610. 10.1007/s10531-015-0948-y

[8] Földvári G, Široký P, Szekeres S, Majoros G, Sprong H. *Dermacentor reticulatus*: a vector on the rise. Parasit Vectors. 2016;9:314. 10.1186/s13071-016-1599-x 27251148 PMC4888597

[9] Jongejan F, Ringenier M, Putting M, Berger L, Burgers S, Kortekaas R, et al. Novel foci of *Dermacentor reticulatus* ticks infected with *Babesia* canis and *Babesia caballi* in the Netherlands and in Belgium. Parasit Vectors. 2015;8:232. 10.1186/s13071-015-0841-2 25889392 PMC4404102

[10] Angelakis E, Waton J, Imbert P, Socolovschi C, Edouard S, Dellamonica P. Scalp eschar and neck lymphadenopathy caused by *Bartonella henselae* after tick bite. 2010;50:549–51. 10.1086/650172 20070235

[11] Dubourg G, Socolovschi C, Del Giudice P, Fournier PE, Raoult D. Scalp eschar and neck lymphadenopathy after tick bite: an emerging syndrome with multiple causes. Eur J Clin Microbiol Infect Dis. 2014;33:1449–56. 10.1007/s10096-014-2090-2 24682865

[12] Duscher GG, Hodžić A, Weiler M, Vaux AGC, Rudolf I, Sixl W, et al. First report of *Rickettsia raoultii* in field collected *Dermacentor reticulatus* ticks from Austria. Ticks Tick Borne Dis. 2016;7:720–2. 10.1016/j.ttbdis.2016.02.022 26976704

[13] Rudolf I, Venclíková K, Blažejová H, Betášová L, Mendel J, Hubálek Z, et al. First report of *Rickettsia raoultii* and *Rickettsia helvetica* in *Dermacentor reticulatus* ticks from the Czech Republic. Ticks Tick Borne Dis. 2016;7:1222–4. 10.1016/j.ttbdis.2016.07.011 27473853

[14] Szekeres S, Docters van Leeuwen A, Rigó K, Jablonszky M, Majoros G, Sprong H, et al. Prevalence and diversity of human pathogenic rickettsiae in urban versus rural habitats, Hungary. Exp Appl Acarol. 2016;68:223–6. 10.1007/s10493-015-9989-x 26613759

[15] Socolovschi C, Mediannikov O, Raoult D, Parola P. Update on tick-borne bacterial diseases in Europe. Parasite. 2009;16:259–73. 10.1051/parasite/2009164259 20092057

